# Appendicular schwannoma presenting as vague abdominal pain

**DOI:** 10.1093/jscr/rjy149

**Published:** 2018-07-03

**Authors:** Abdullah Mohammed Alshamrani, Rami Abdulrahman Sairafi, Ali Mohammed Alzahrani, Mostafa Abdel-Raheem

**Affiliations:** General Surgery Department, Security Forces Hospital Program, Riyadh, Saudi Arabia

## Abstract

Appendicular schwannomas are very rare condition with nonspecific clinical symptoms and frequently recognized during pathological examination. They arise less frequently in the gastrointestinal tract and comprise ~1% of all malignant gastrointestinal tumours. We presented a rare case of an appendicular schwannoma that was discovered incidentally in a 25-year-old student diagnosed with appendicular mucocoele with a suspected obstructing tumoural lesion based on computed tomography findings. A colonoscopy examination showed a bulging, nodular, erythematous lesion at the base of the caecum (appendiceal orifice). Biopsies showed mixed inflammatory infiltration in the lamina propria, with lymphoid-filled formations. No evidence of dysplasia or neoplasia. Tumour markers were negative. Appendicular neoplasms, such as schwannomas of the appendix, are rarely associated with nonspecific clinical symptoms and are frequently recognized during pathological examination of the resected appendix. Laparoscopic surgery with a clear resection margin is the cornerstone of treatment for appendicular schwannoma, and it is associated with a favourable prognosis.

## INTRODUCTION

Schwannomas are benign neurogenic tumours that arise in the Schwann cells, which form a sheath around the peripheral nerves, providing support and protection. In most instances, schwannomas occur in the head, neck, upper and lower limbs as well as the third to twelfth cranial nerves. They arise less frequently in the gastrointestinal tract (mainly the stomach and small intestine) and comprise ~1% of all malignant gastrointestinal tumours [1]. A few reports have described a case of appendicular schwannoma [2–6], with some patients presenting clinical symptoms and signs typical of acute appendicitis [4, 5]. This report describes the case of a patient who had an unusual clinical course, including an unusually long symptom duration and vague right iliac fossa pain.

## CASE REPORT

A 25-year-old dental student was referred to our institution for the management of vague abdominal pain in the right lower quadrant for a duration of 5 years. The pain was colicky, intermittent and relieved by analgesia. It was associated with nausea, vomiting and anorexia. The primary diagnosis was mucocoele of the appendix with suspected obstruction by a tumoural lesion based on an abdominal computed tomography (CT) scan.

The patient had no history of fever or change in bowel habits. There was also no history of weight loss, fatigue, night sweats or previous attacks of abdominal pain, no family history of a similar clinical picture and no history of malignancy. His medical and surgical history were irrelevant.

On examination, the patient was conscious, alert and oriented and not in pain. His vital signs were as follows: heart rate of 88 beats per minute, respiratory rate of 22 cycles per minute, blood pressure of 123/86 mmHg and oxygen saturation of 98%. A digital rectal examination revealed soft stool in the rectum.

After an initial examination by a colorectal surgeon, the patient was referred for a colonoscopy. Further investigations included laboratory workup, histopathology, an abdominal X-ray and a CT scan of the abdomen. The patient was offered laparoscopic-assisted ileocecal resection following the results of the investigations.

Blood was drawn for a complete blood count, urea and electrolytes, liver function test, coagulation profile, serum glucose level, carcinoembryonic antigen and cancer antigen, which were all within normal limits.

**Figure 1:**
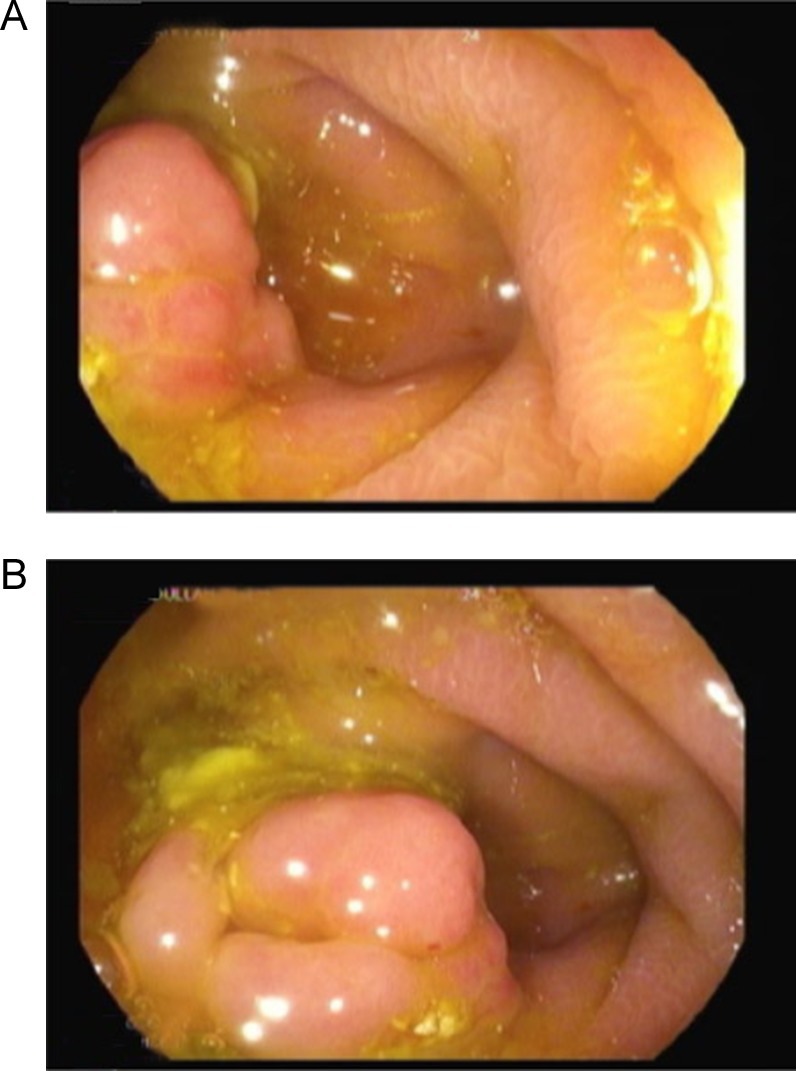
Colonoscopy images showing a bulging, erythematous nodular lesion at the base of the caecum.

**Figure 2:**
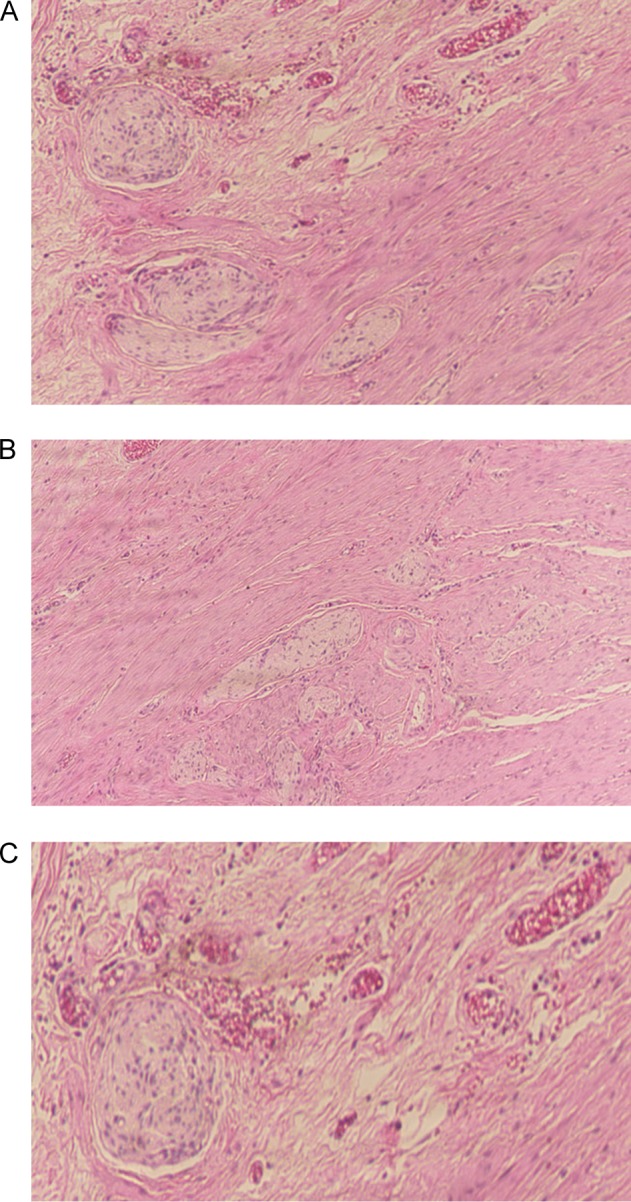
Histopathological examination of the appendiceal specimen revealed hypertrophied bundles within lamina propria consistent with appendiceal schwannoma (**A**, **B**, **C**).

The colonoscopy revealed a bulging, erythematous nodular lesion at the base of the caecum (appendiceal orifice) (Fig. 1A, B). Samples were obtained from the lesion and sent for histopathological analysis. The examination showed mixed inflammatory infiltration in the lamina propria with a lymphoid-filled formation (Fig. 2). No dysplasia or neoplasia was observed, and the tumour was negative for markers. Small piles were seen; however, no other gross abnormalities were observed.

**Figure 3:**
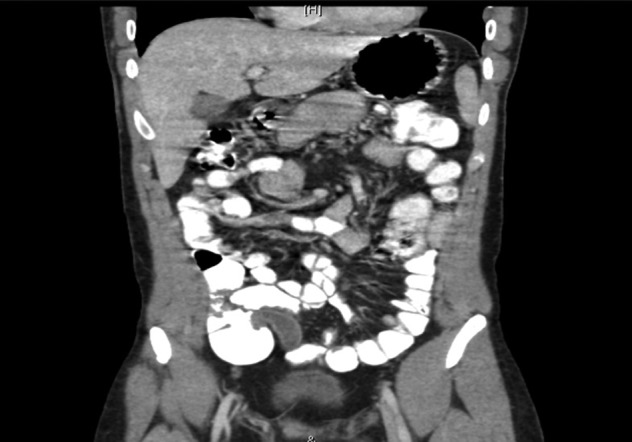
A computed tomography scan of the abdomen showing a dilated appendix with no surrounding fat stranding or free fluid. The wall of the appendix was not interrupted. The liver, spleen, adrenal glands, kidneys, pancreas appeared unremarkable. No free air or free fluid was noted in the abdomen or pelvis. Overall, the findings were consistent with sub-acute appendicitis.

The abdominal X-ray was reported as normal. The abdominal CT scan revealed a dilated appendix measuring 1.6 cm in maximum diameter with no surrounding fat stranding or free fluid. No free air was noted. The wall of the appendix was not interrupted. The liver showed homogeneous enhancement with no focal lesion (Fig. 3). No intrahepatic biliary duct dilatation was observed, and the portal vein and hepatic veins were patent. The spleen, adrenal glands, kidneys and pancreas appeared unremarkable. No free air or free fluid was noted in the abdomen or pelvis. The lung bases and bony skeleton also appeared unremarkable. The findings were consistent with sub-acute appendicitis.

After the laboratory and radiological investigations, the patient underwent laparoscopic-assisted ileocecal resection with side-to-side anastomosis.

A repeat histopathological examination, performed after surgery, showed hypertrophied bundles within the lamina propria, consistent with appendiceal schwannoma. No evidence of malignancy was identifiable.

## DISCUSSION

The rarity and unusual clinical presentation of appendicular schwannomas may contribute to underreporting because there are overlapping features with several other tumours and inflammatory conditions. In about 30–50% of appendicular tumours, patients present clinical symptoms and signs typical of appendicitis [7, 8]. However, for most patients, the symptoms are nonspecific, making the preoperative diagnosis challenging. Our patient had an unusual clinical course characterized by chronic right iliac fossa pain for a duration of 5 years, and a definitive diagnosis was only reached 6 months after the patient’s initial presentation to our institution.

As demonstrated in our case, conventional imaging modalities, such as CT and endoscopy, are useful in determining tumour location. Other investigational modalities that can help identify tumour location include ultrasound, barium enema and magnetic resonance imaging [9]. In some cases, mesenteric vessel angiography can reveal hypervascularized tumour vessels. In our case, we could not determine the nature of the tumour by using conventional imaging techniques. An initial abdominal CT scan suggested an appendicular mucocoele, whereas a second scan revealed findings consistent with sub-acute appendicitis. Although a histopathological analysis permitted us to rule out malignancy, the diagnosis of appendicular schwannoma was only reached after repeat histology. It is possible that inadequate tissue acquisition may have contributed to the missed diagnosis.

The only treatment option that was explored in our patient was laparoscopic-assisted ileocecal resection with a clear margin 5 cm proximal and 5 cm distal, which was also offered in other reported cases and is considered the treatment of choice in these patients [3–5, 10]. The surgical approach varies depending on factors such as tumour size and histologic characteristics, which determine the prognosis. However, there is no consensus regarding a specific surgical approach.

In conclusion, we presented a rare case of an appendicular schwannoma that was discovered incidentally in a 25-year-old patient diagnosed with appendicular mucocoele. Appendicular neoplasms, such as schwannomas of the appendix, are rarely associated with nonspecific clinical symptoms and are frequently recognized during pathological examination of the resected appendix. Laparoscopic surgery with a clear resection margin is the cornerstone of treatment for appendicular schwannoma, and it is associated with a favourable prognosis.

## ABBREVIATION

CT,: computed tomography scan

## References

[1] Melvin WS, Wilkinson MG Gastric schwannoma. Clinical and pathologic considerations. Am Surg 1993;59:293–6. 8489097

[2] Gupta A, Rattan KN, Gupta A, Banerjee D Giant appendicular schwannoma in a child. Indian J Pathol Microbiol 2009;52:281. 19332945 10.4103/0377-4929.48950

[3] Suh SW, Park JM, Choi YS, Cha SJ, Chang IT, Kim BG Laparoscopic approach to a case of appendicular schwannoma. J Korean Soc Coloproctol 2010;26:302–6. 21152234 10.3393/jksc.2010.26.4.302 PMC2998005

[4] Kalaycı M, Akyüz Ü, Demirağ A, Gürses B, Özkan F, Gökçe Ö Retroperitoneal Schwannoma: A Rare Case [Internet]. Case Reports in Gastrointestinal Medicine. 2011 [cited 2018 Mar25]. Available from: https://www.hindawi.com/journals/crigm/2011/465062/10.1155/2011/465062 PMC3350261 22606418

[5] Kamp MC, Unen JMJ Appendicular schwannoma presenting as acute appendicitis. Acta Chir Belg 2015;115:317–8. 26324037 10.1080/00015458.2015.11681120

[6] Cheng E, Oliphant R, Fung C, Rickard M, Keshava A Schwannoma of the appendix: a case report and review of the literature. Surg Pract [Internet]. 2017 Dec 28 [cited 2018 Mar 25]; Available from: https://onlinelibrary.wiley.com/doi/abs/10.1111/1744-1633.12290

[7] Checkoff JL, Wechsler RJ, Nazarian LN Chronic inflammatory appendiceal conditions that mimic acute appendicitis on helical CT. AJR Am J Roentgenol 2002;179:731–4. 12185054 10.2214/ajr.179.3.1790731

[8] Daly CP, Cohan RH, Francis IR, Caoili EM, Ellis JH, Nan B Incidence of acute appendicitis in patients with equivocal CT findings. AJR Am J Roentgenol 2005;184:1813–20. 15908536 10.2214/ajr.184.6.01841813

[9] Lee NJ, Hruban RH, Fishman EK Abdominal schwannomas: review of imaging findings and pathology. Abdom Radiol N Y 2017;42:1864–70. 10.1007/s00261-017-1088-5 28265705

[10] Tang S-X, Sun Y-H, Zhou X-R, Wang J Bowel mesentery (meso-appendix) microcystic/reticular schwannoma: case report and literature review. World J Gastroenterol 2014;20:1371–6. 24574814 10.3748/wjg.v20.i5.1371 PMC3921522

